# Identification of Crucial Cancer Stem Cell Genes Linked to Immune Cell Infiltration and Survival in Hepatocellular Carcinoma

**DOI:** 10.3390/ijms252211969

**Published:** 2024-11-07

**Authors:** Lien-Hung Huang, Shao-Chun Wu, Yueh-Wei Liu, Hang-Tsung Liu, Peng-Chen Chien, Hui-Ping Lin, Chia-Jung Wu, Ting-Min Hsieh, Ching-Hua Hsieh

**Affiliations:** 1 Department of Plastic Surgery, Kaohsiung Chang Gung Memorial Hospital and Chang Gung University College of Medicine, Kaohsiung 833, Taiwan; ahonbob@gmail.com (L.-H.H.); venu_chien@hotmail.com (P.-C.C.); poppy952@gmail.com (H.-P.L.); alice8818@yahoo.com.tw (C.-J.W.); 2 Department of Anesthesiology, Kaohsiung Chang Gung Memorial Hospital and Chang Gung University College of Medicine, Kaohsiung 833, Taiwan; shaochunwu@gmail.com; 3 Department of General Surgery, Kaohsiung Chang Gung Memorial Hospital and Chang Gung University College of Medicine, Kaohsiung 833, Taiwan; anthony0612@adm.cgmh.org.tw; 4 Department of Trauma Surgery, Kaohsiung Chang Gung Memorial Hospital and Chang Gung University College of Medicine, Kaohsiung 833, Taiwan; htl1688@yahoo.com.tw

**Keywords:** hepatocellular carcinoma, cancer stem cells, mRNA expression-based stemness index, cell cycle genes, tumor microenvironment

## Abstract

Hepatocellular carcinoma is characterized by high recurrence rates and poor prognosis. Cancer stem cells contribute to tumor heterogeneity, treatment resistance, and recurrence. This study aims to identify key genes associated with stemness and immune cell infiltration in HCC. We analyzed RNA sequencing data from The Cancer Genome Atlas to calculate mRNA expression-based stemness index in HCC. A weighted gene co-expression network analysis was performed to identify stemness-related gene modules. A single-sample gene set enrichment analysis was used to evaluate immune cell infiltration. Key genes were validated using RT-qPCR. The mRNAsi was significantly higher in HCC tissues compared to adjacent normal tissues and correlated with poor overall survival. WGCNA and subsequent analyses identified 10 key genes, including minichromosome maintenance complex component 2, cell division cycle 6, forkhead box M1, NIMA-related kinase 2, Holliday junction recognition protein, DNA topoisomerase II alpha, denticleless E3 ubiquitin protein ligase homolog, maternal embryonic leucine zipper kinase, protein regulator of cytokinesis 1, and kinesin family member C1, associated with stemness and low immune cell infiltration. These genes were significantly upregulated in HCC tissues. A functional enrichment analysis revealed their involvement in cell cycle regulation. This study identified 10 key genes related to stemness and immune cell infiltration in HCC. These genes, primarily involved in cell cycle regulation, may serve as potential targets for developing more effective treatments to reduce HCC recurrence and improve patient outcomes.

## 1. Introduction

Hepatocellular carcinoma (HCC) is the most common type of primary liver cancer, accounting for nearly 90% of all occurrences. Cirrhosis, hepatitis B virus or hepatitis C virus infection, and chronic alcohol abuse are the leading causes of liver cancer. Other risk factors for liver cancer include aflatoxin B1 consumption and metabolic syndrome [1]. HCC has significant intratumor and interpatient heterogeneity. Interpatient heterogeneity is associated with individualized therapy, and intratumor heterogeneity has a significant impact on the efficacy of medicines in individuals [2]. Intratumor heterogeneity contributes to the difficulty of treating HCC, resulting in poor patient outcomes and survival [3]. Up to 50 to 70% of patients may develop hepatic recurrence after 5 years. Furthermore, 70% of patients with recurrent HCC have an early relapse within two years of surgery, which is largely incurable and has been associated with a poor outcome [4]. Among the various factors contributing to tumor refractory, the presence of cancer stem cells (CSCs) has been notorious.

CSCs account for less than 1% of all tumor cells and are characterized by their ability to self-renew and differentiate [5,6]. CSCs are formed at the time of tumor onset from differentiated cells or adult tissue-resident stem cells [7]. CSCs are assumed to be responsible for generating a heterogeneous tumor lesion, and contributing to treatment resistance, tumor relapse, metastasis, and the avoidance of immunological surveillance [8,9]. CSCs escape multiple drug actions through various mechanisms, including the activation of DNA repair pathway, high expression of drug efflux-related proteins, the ability to reconstitute original tumors, and activation of signaling pathways involved in epithelial–mesenchymal transition, hypoxia stimulation, and abnormal angiogenesis [10,11,12]. Recently, several strategies have been developed with the specific goal of eradicating CSCs and their niche [13]. The search for biomarkers that characterize these CSCs and enable therapeutic and prognostic prediction or tracking is necessary. Numerous surface and intracellular biomarkers have been evaluated for CSC purification and correlated with diagnosis, treatment, and prognosis in various cancer cells [14].

In HCC, a number of markers for liver CSCs have been identified, including CD13, CD24, CD44, CD47, CD90, CD133, epithelial cell adhesion molecule (EpCAM), oval cell marker, delta-like non-canonical Notch ligand 1, keratin 19, ATP-binding cassette super-family G member 2, aldehyde dehydrogenase 1, etc. [10,15,16]. Several clinical trials have been launched for cancer treatments targeting CSC-specific surface markers or stemness-related pathways [16,17]. For example, catumaxomab, an FDA-approved chimeric antibody that binds to antigens CD3 and EpCAM, is used in the treatment of malignant ascites.

Machine learning is already used effectively in several areas, such as diagnosing cancer, predicting patient outcomes, and informing treatment planning [18]. Stem features extracted from transcriptome and epigenetic signatures can also be achieved through machine learning [19]. The mRNA expression-based stemness index (mRNAsi) reflects cancer stemness was calculated by analyzing RNA sequencing data, and the epigenetic regulation based-index (EREG-mRNAsi) was calculated by the epigenetic regulation features learned through a one-class logistic regression (OCLR) algorithm [19]. These stem cell indexes provide ideas for exploring genes related to the existence and maintenance of CSCs in a variety of cancers and discovering the unanticipated biomarkers of CSCs [20,21,22].

In this study, we aimed to establish the stemness-associated CSC genes for HCC. RNA sequencing data for HCC recurrence tissue were obtained from The Cancer Genome Atlas (TCGA) database. We evaluated the association between the mRNAsi and HCC. Next, the differential expression genes (DEGs) between HCC and adjacent normal liver tissues were analyzed through weighted gene co-expression network analysis (WGCNA). Using univariate Cox regression analysis, we screened out mRNAsi-related genes. After survival analysis, 25 key genes were identified. We further evaluated the potential role of 25 key genes in immune cell infiltration by single-sample gene set enrichment analysis (ssGSEA). Finally, we identified 10 stemness-associated CSCs genes, including cell division cycle 6 (CDC6), denticleless E3 ubiquitin protein ligase homolog (DTL), forkhead box M1 (FOXM1), Holliday junction recognition protein (HJURP), kinesin family member C1 (KIFC1), minichromosome maintenance complex component 2 (MCM2), maternal embryonic leucine zipper kinase (MELK), NIMA-related kinase 2 (NEK2), protein regulator of cytokinesis 1 (PRC1), and DNA topoisomerase II alpha (TOP2A). Functional enrichment analysis revealed that these genes are involved in cell cycle regulation. Key genes were validated using RT-qPCR in prospectively collected HCC samples vs. adjacent liver tissues in 20 individuals.

## 2. Results

### 2.1. mRNAsi and EREG-mRNAsi in HCC

**Figure 1:**
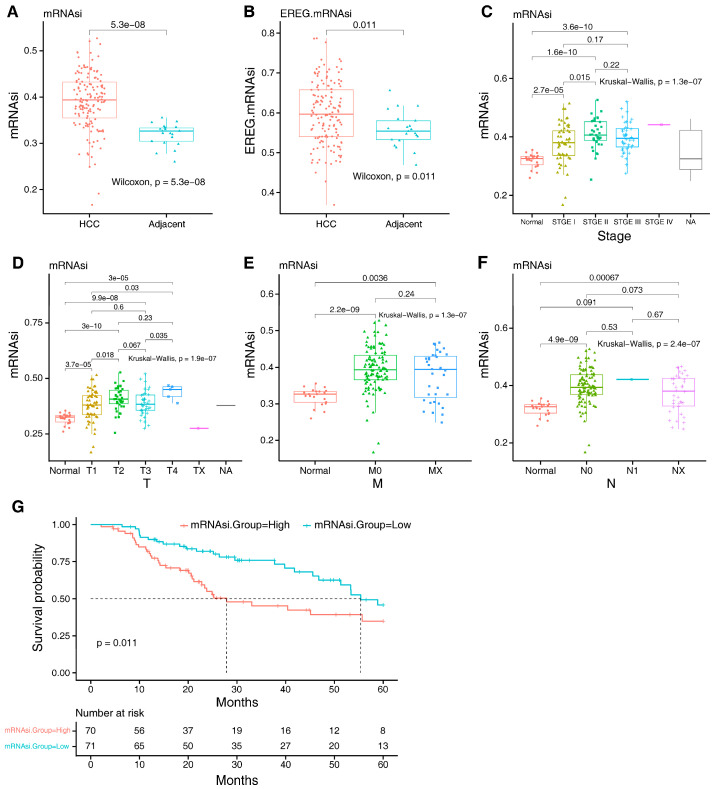
Relationships between the mRNAsi and the clinicopathological characteristics and prognosis of HCC patients. (**A**) mRNAsi score and (**B**) EREG-mRNAsi score in HCC group and adjacent normal group. (**C**) Relationship between mRNAsi and clinical stage. (**D**–**F**) Relationship between mRNAsi and tumor status. (**G**) Kaplan–Meier analysis of the relationship between mRNAsi and overall survival of HCC. mRNAsi: mRNA expression-based stemness index; EREG-mRNAsi: epigenetic regulation based-index.

mRNAsi and EREG-mRNAsi are important indexes to evaluate the overall stemness of tumor cells. The mRNAsi and EREG-mRNAsi scores varied from 0 to 1 based on the OCLR method, representing stemless and stemness. The mRNAsi and EREG-mRNAsi were compared between 141 HCC tissues and 21 adjacent normal tissues. The mRNAsi and EREG-mRNAsi were higher in the HCC group than in the adjacent normal group (Figure 1A,B). We also evaluated the correlation between the mRNAsi and clinicopathological characteristics. The mRNAsi values for the pathologic tumor stage I group were significantly lower than the stage II group (*p* < 0.05). However, no difference in the mRNAsi values was seen between the stages I and III groups (Figure 1C). The pathologic T1 stage had considerably lower mRNAsi values compared to the pathologic T2 and T4 stages (*p* < 0.05); however, there was no difference with the T3 stage (Figure 1D). The difference in mRNAsi values was not observed for different pathologic N or M stages. (Figure 1E,F). The results showed that the mRNAsi did not have a significant correlation with the tumor stage of HCC. Next, a Kaplan–Meier curve was used to assess the impact of mRNAsi values on the prognosis of HCC patients. Patients with lower mRNAsi values had prolonged overall survival (*p* = 0.011) (Figure 1G). These results indicated that the mRNAsi is significantly related to the occurrence and overall survival of HCC.

### 2.2. mRNAsi Evaluated in the Context of the Tumor Mutation

**Figure 2:**
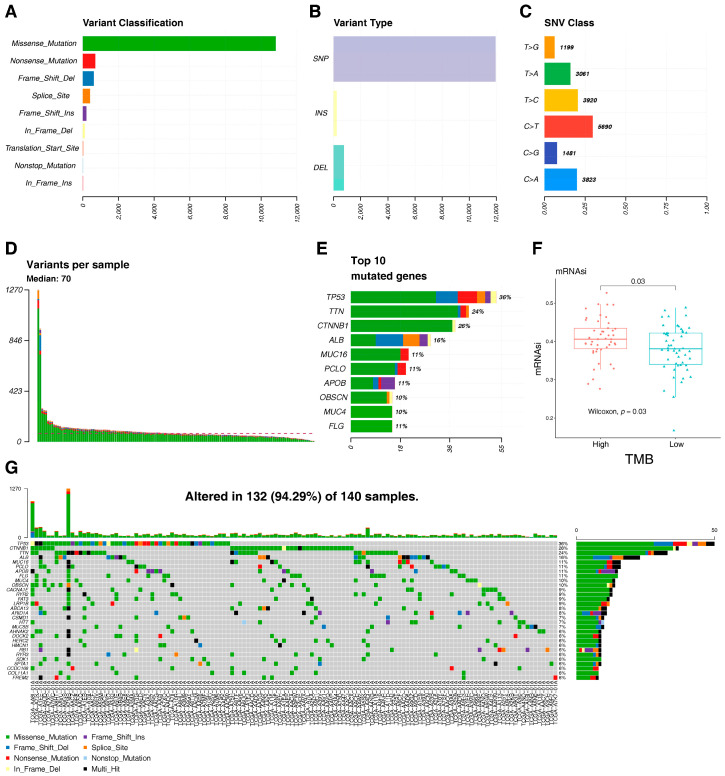
Summary of the TMB information. (**A**) Missense mutation was the most common variant classification and had the highest frequency. (**B**) SNP occurred most frequently in variant types. (**C**) C > T accounted for the most fraction in SNV. (**D**) The number of tumor mutation burdens in specific samples. (**E**) The top 10 mutated genes in HCC. (**F**) Relationship between mRNAsi and TMB. (**G**) Landscape of mutation profiles in HCC. SNP: single-nucleotide polymorphism; INS: insertion; DEL: deletion; SNV: single-nucleotide variant.

We further analyzed the relationship between the mRNAsi and the mutation profiles of HCC with the expression of tumor mutational burden (TMB), a numeric index that expresses the number of mutations per megabase (muts/Mb) carried by tumor cells in a neoplasm [23]. The results showed that missense mutations, single-nucleotide polymorphisms, and C > T transitions predominated among the variant classifications, variant types, and SNV categories of HCC, respectively (Figure 2A–C). The number of altered bases from each patient is shown in Figure 2D. According to the mutation frequency, the top 10 mutated genes were displayed in Figure 2E, including *TP53* (36%), *TTN* (24%), *CTNNB1* (26%), *ALB* (16%), *MUC16* (11%), *PCLO* (11%), *APOB* (11%), *OBSCN* (10%), *MUC4* (10%), and *FLG* (11%). A waterfall plot was used to illustrate the mutation information for the top 30 mutated genes in each patient (Figure 2G). These genes were mutated in 94.29% of HCC. Finally, we analyzed the correlations between somatic mutations in the top 10 mutated genes and the mRNAsi. The result showed that the mRNAsi values of the high TMB group were significantly higher than those of the low TMB group (Figure 2F). These results indicated that the mRNAsi is related to the tumor mutation of HCC.

### 2.3. Identification of mRNAsi-Related Key Genes by WGCNA

**Figure 3:**
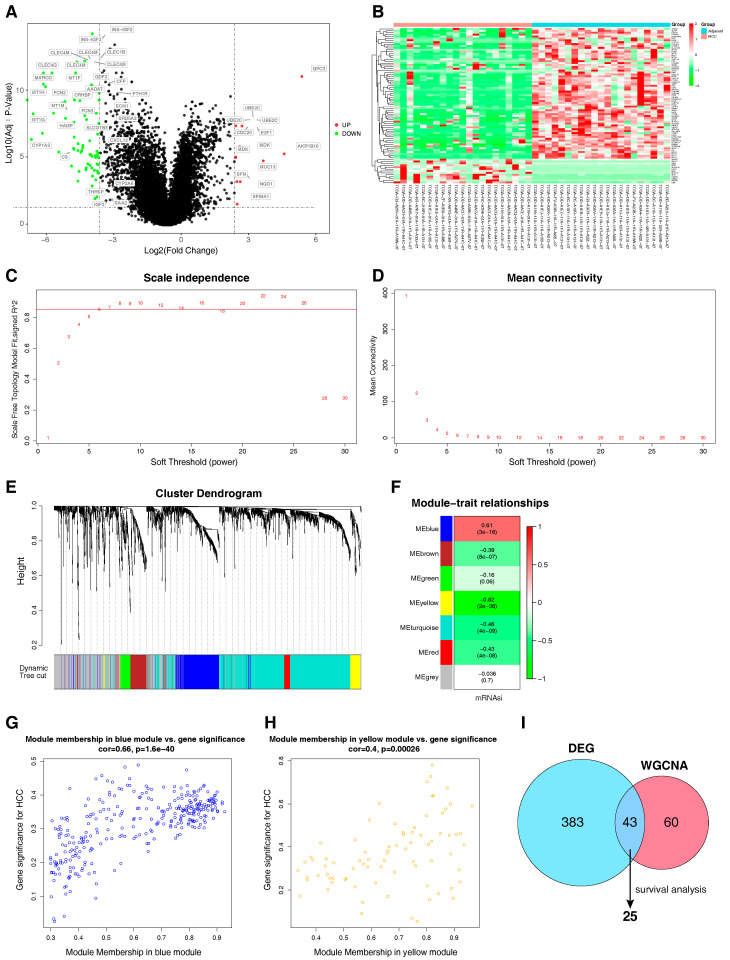
The mRNAsi index associated with WGCNA of HCC. (**A**,**B**) A volcano plot and heat map of DEGs in HCC; green indicates downregulated genes and red indicates upregulated genes. |Log2FoldChange| >3, *p* < 0.05. (**C**,**D**) Determination of soft threshold for the similarity matrix. The scale-free correlation coefficient and the mean connectivity for soft threshold powers were analyzed. The number represents the power value and the horizontal axis represents the soft threshold power = 6. (**E**,**F**) Gene clustering and gene module partition results. The different branches of the cluster dendrogram correspond to different gene modules that are represented by different colors. (**G**,**H**) The correlation between the gene modules and mRNAsi. The Pearson correlation coefficient of the gene module and the traits was plotted as a heat map. (**I**) The intersection of the DEGs and mRNAsi-related WGCNA-derived genes. DEG: differential expression genes; WGCNA: weighted gene co-expression network analysis.

To construct the mRNAsi-related modules by WGCNA, the differential analysis was first performed using RNA sequencing data from 21 HCC tissues and 21 adjacent normal tissues. From this analysis, a total of 2799 DEGs (|log2 fold change| > 1, *p* < 0.05) were screened. We extracted the top 108 DEGs (|log2 fold change| > 3, *p* < 0.05) and visualized them in a volcano plot (Figure 3A) and heat map (Figure 3B). Then, WGCNA was used to analyze the filtered DEGs to construct a co-expression network (Figure 3C,D). The most critical parameter of the soft threshold power was set at 6 to ensure the integral connectivity of co-expression modules. The distribution of genes in every module was visualized as shown in (Figure 3E). There were seven modules in the WGCNA results, and the blue module (0.61, *p* < 0.05) and the yellow module (–0.82, *p* < 0.05) were closely related to the mRNAsi (Figure 3F). Then, we investigated the key genes that were highly associated with HCC in each module, using the threshold values Module Membership > 0.8 and Gene Significance > 0.3 (Figure 3G,H).

As a result, we adopted 103 genes from blue and yellow modules as the target genes. To narrow down the target genes, we improved the criteria of DEGs (|log2 fold change| > 2, *p* < 0.05) and intersected them with 103 target genes. A total of 43 target genes were identified for subsequent studies (Figure 3I). Next, we displayed the survival curve for each gene and obtained 25 key genes closely related to the survival of HCC (Supplemental Figure S1).

### 2.4. The Immune Cell Infiltration Analysis by ssGSEA

**Figure 4:**
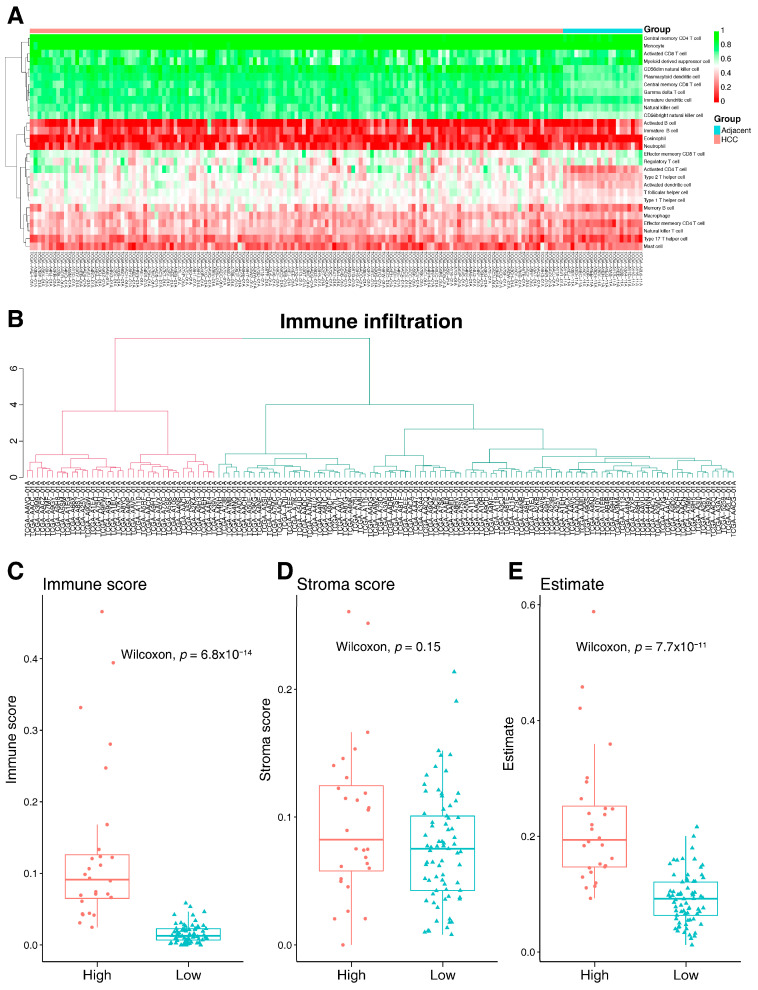
The immune cell infiltration level was inferred using ssGSEA. (**A**) Derived immune cell infiltration scores using 28 immune cell types. (**B**) Classifying samples into two groups (cluster 1 and cluster 2) using unsupervised learning. (**C**–**E**) Differences in immune score, stromal score, and ESTIMATE score between clusters 1 and 2.

**Figure 5:**
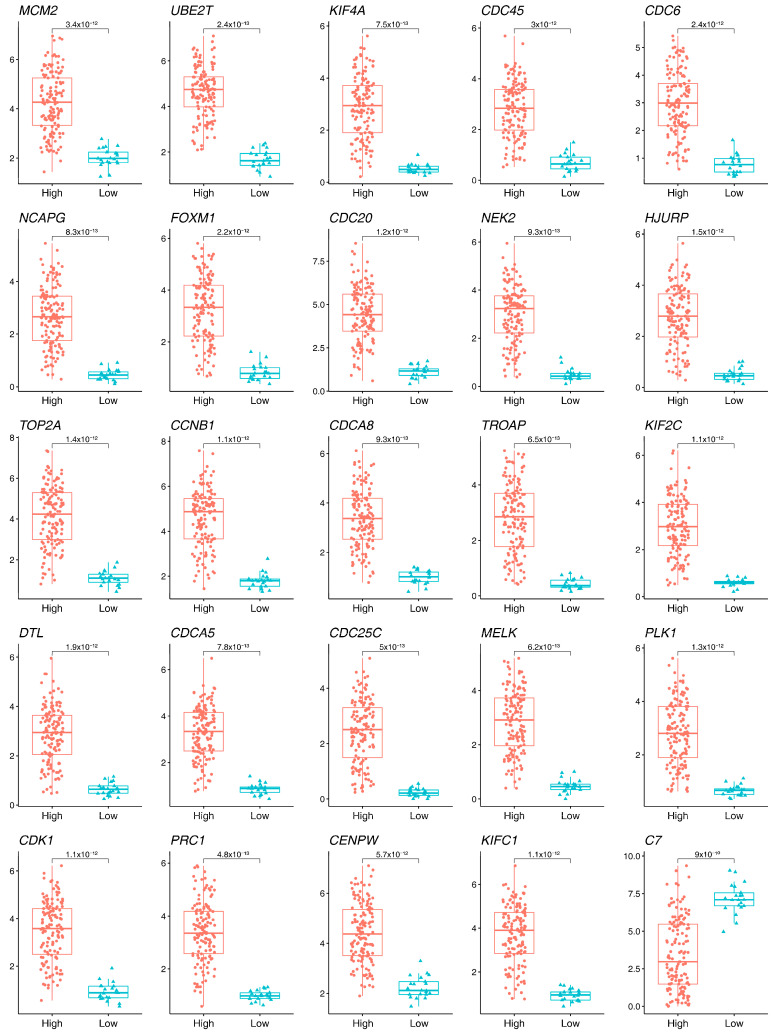
Differences in 25 key genes expression between different clusters. A total of 10 out of 25 key genes were significantly upregulated in the low group.

To evaluate the potential role of 25 key genes in the tumor immune microenvironment, the immune cell infiltration level of 28 immune cell types was inferred using ssGSEA (Figure 4A). According to the unsupervised learning, the 141 HCC samples were clustered into two groups (cluster 1 and cluster 2) (Figure 4B). The samples in cluster 1 were characterized by a significantly higher immune score and ESTIMATE score, but the stromal score did not increase significantly (Figure 4C–E). This result revealed that the samples in cluster 1 had a higher level of immune cell infiltration. Cluster 2 showed a reduced connection with immune infiltration. As a result, cluster 1 was designated as the “high” group, whereas cluster 2 was designated as the “low” group. We further investigated the 25 key gene expressions between the high and low groups. As a result, the expression of 10 genes was significantly upregulated in the low group (Figure 5), including *CDC6*, *DTL*, *FOXM1*, *HJURP*, *KIFC1*, *MCM2*, *MELK*, *NEK2*, *PRC1*, and *TOP2A*. This result indicated that these 10 key genes were associated with immune cell infiltration in HCC.

### 2.5. Validation and Functional Enrichment Analysis of Key Genes

**Figure 6:**
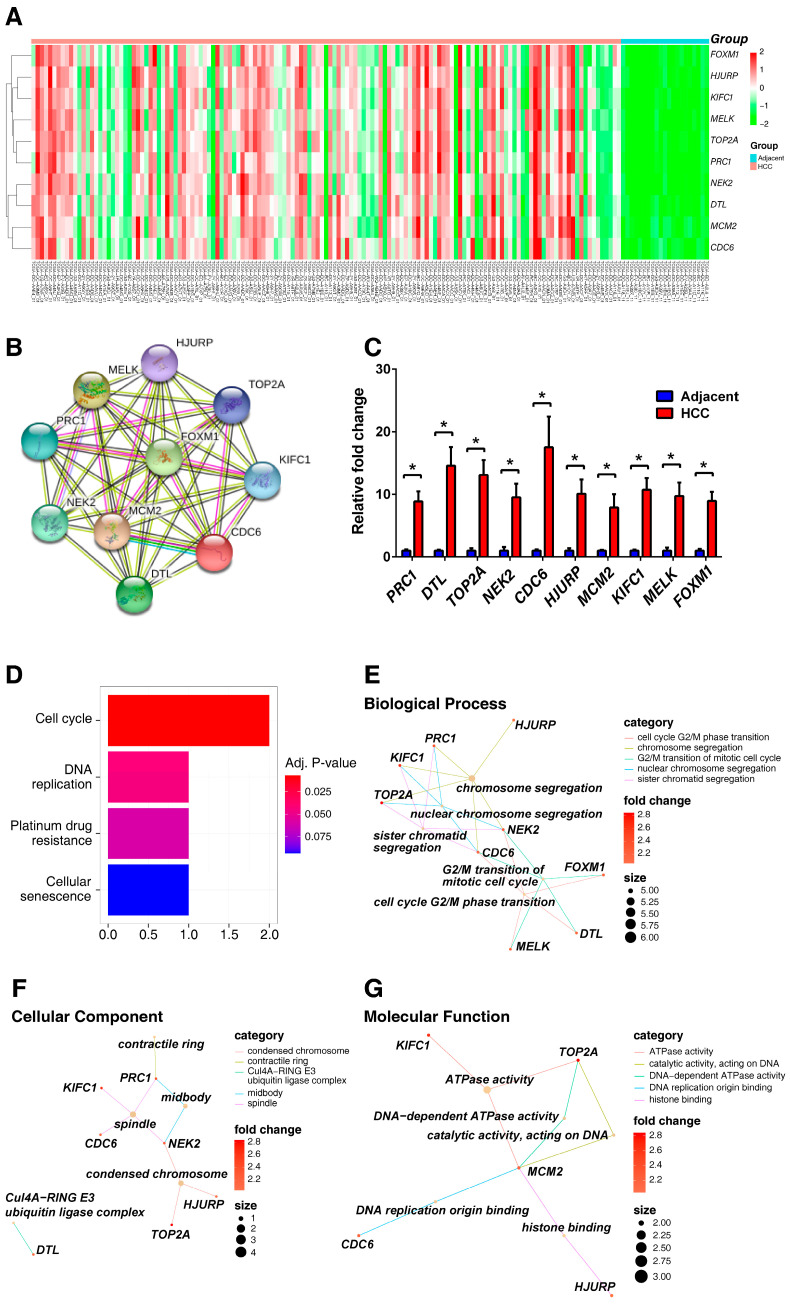
Functional enrichment analysis of 10 key genes in HCC. (**A**) The clustered heat map in HCC and adjacent normal tissue. (**B**) PPI networks were performed for the key genes by STRING. (**C**) RT-qPCR was performed to measure the expression of 10 key genes. The enrichment analysis of the 10 key genes of (**D**) KEGG pathway, (**E**) biological process, (**F**) cellular component, and (**G**) molecular function. * indicated a significance of *p* < 0.05.

A heat map depicted the expression levels of ten critical genes in 141 HCC and 21 adjacent normal tissues (Figure 6A). Real-time qPCR was used to determine the expression levels of 10 key genes (20 adjacent normal and 20 HCC samples). Figure 6C demonstrates that all 10 key genes were significantly upregulated in HCC tissue. In addition, the expression of 10 key genes was significantly higher in the advanced stage of HCC (stage III) than in the early stage (stage I+II) (Table 1). These results indicated that these 10 key genes were associated with the pathological stage of HCC. To explore the interactions between the 10 key genes, we developed protein–protein interaction networks by STRING. As a result, the 10 nodes and 44 edges were formed in the network (Figure 6B). These genes were biologically connected as a group. Subsequently, we performed Kyoto Encyclopedia of Genes and Genomes (KEGG) pathway enrichment analysis. As shown in Figure 6D, the most distinguished pathway was related to the cell cycle. We further performed a gene ontology analysis of 10 key genes. For biological processes, the key genes were mainly enriched in chromosome segregation and cell cycle G2/M phase transition (Figure 6E). In the cellular component, key genes are enriched in spindles, condensed chromosomes, and the midbody (Figure 6F). In molecular function, the key genes were associated with ATPase activity, DNA-dependent ATPase activity, catalytic activity, and acting on DNA (Figure 6G). Functional enrichment analysis revealed that cell cycle regulation may be relevant for HCC cancer cell stemness pathways.

## 3. Discussion

HCC is an aggressive disease with poor outcomes. According to the CSC concept, tumor growth is driven by a subpopulation of tumor stem cells within malignancies. This concept explains clinical data in HCC and other cancers, such as tumor recurrence after chemotherapy or radiotherapy and treatment resistance [17]. Understanding and targeting CSCs is essential for developing more effective treatments that can reduce the high recurrence rate of HCC, ultimately improving outcomes for HCC patients [24].

The mRNAsi is a metric used in cancer research to quantify the stemness of tumor cells based on their gene expression profiles. mRNAsi is used to identify genes associated with tumor stemness in many cancers, including gastric cancer [25], breast cancer [26], pancreatic ductal adenocarcinoma [27], and HCC [28]. In this study, we explored the difference between the mRNAsi in HCC samples and adjacent normal samples, and their relationship to the clinicopathological characteristics of HCC patients. As expected, HCCs had significantly higher mRNAsi than adjacent normal tissues. Patients with the high mRNAsi scores have lower survival rates than patients with low mRNAsi scores. These results were consistent with the current studies that show that an increase in tumor stemness was closely associated with the poor prognosis and the disease progression of HCC patients [29,30,31]. Furthermore, we established that mRNAsi is associated with tumor mutations in HCC using TMB analysis. The high TMB group had considerably higher mRNAsi values than the low TMB group. Current studies have shown that there can be a positive correlation between TMB and mRNAsi in certain cancers [25,32]. High-risk HCC patients with a poor prognosis had a higher TMB and mRNAsi than those in the low-risk group [33].

Based on WGCNA mining, for the gene modules closely related to mRNAsi and subsequent ssGSEA analysis, we obtained 10 key genes related to immune cell infiltration in HCC, including *CDC6*, *DTL*, *FOXM1*, *HJURP*, *KIFC1*, *MCM2*, *MELK*, *NEK2*, *PRC1*, and *TOP2A*. These 10 key genes were associated with the pathological stage of HCC. MCM2 is an important regulator of DNA replication. The dysfunction of MCM2 results in the occurrence and progression of multiple cancers [34]. The expression of MCM2 in HCC tissue is higher than in normal liver tissue. MCM2 enhances the stemness of HCC cells, while the downregulation of MCM2 inhibits resistance towards sorafenib [35]. CDC6 plays an important role in DNA replication and cell cycle regulation. The dysregulation of CDC6 may negatively impact genome integrity and induce malignant cell proliferation [36]. The expression levels of CDC6 are higher in HCC tissues compared to adjacent normal tissues. The expression of CDC6 is closely related to the infiltrating levels of CD8^+^ T cells, CD4^+^ T cells, macrophages, neutrophils, and dendritic cells in HCC tissues [36]. FOXM1 is a proliferation-associated transcription factor that plays a critical role in cancer development and progression. FOXM1 is a master regulator of the G1–S and G2–M phases of the cell cycle and mitotic spindle integrity [37]. FOXM1 is overexpressed in human HCC tissues and is an important risk factor for HCC recurrence and survival [38]. NEK2 is a member of the NEK serine/threonine kinase and plays a significant role in cell cycle progression in the interphase and M phase. NEK2 enhances HCC metastasis and is correlated with recurrence [39]. HJURP, a chaperone protein of centromere protein A, is upregulated in HCC and associated with poor survival in patients [40]. TOP2A is a topoisomerase IIα that regulates DNA synthesis, transcription, and chromosome segregation during mitosis. A high expression of TOP2A in HCC is associated with disease progression and metastasis [41]. DTL, also known as CDT2 (chromatin licensing and DNA replication factor 2), regulates CDT1 degradation following DNA damage. DTL is overexpressed in HCC, and high levels of DTL expression are associated with poor clinical outcomes and increased somatic mutation rates [42]. MELK modulates intracellular signaling and influences various cellular and biological processes, including the cell cycle. MELK is highly overexpressed in HCC and its overexpression strongly correlates with early recurrence and poor survival in patients. MELK siRNA decreased HCC cell proliferation, invasion, stemness, and tumorigenicity via triggering apoptosis and mitosis [43]. PRC1 is involved in cytokinesis and plays key roles in microtubule organization. PRC1 is upregulated in HCC and its expression correlates with early HCC recurrence. PRC1 exerts oncogenic effects by promoting cancer proliferation, stemness, metastasis, and tumorigenesis [44]. KIFC1 belongs to the C-type kinesin of the kinesin-14 family and plays important roles in intracellular transport and cell division. KIFC1 is highly overexpressed in HCC and positively associated with advanced stages and poor prognosis [45]. In summary, most of these key genes of HCC in this study are closely related to cancer progression, metastasis, cancer recurrence, and cell cycle regulation of HCC. It is consistent with functional enrichment analysis, which shows that these genes are mainly involved in the cell cycle.

Through ssGSEA, we found that these 10 key genes were significantly highly expressed in the low immune cell infiltration group. Current research demonstrated that the HCC group with the best prognosis had higher CD4^+^ T cell infiltration. The HCC group with the worse prognosis had a low CD8^+^/regulatory T cell ratio [46]. HCC exhibits reduced immune cell infiltration, which may be partly due to cell cycle-influenced changes in the tumor microenvironment.

## 4. Materials and Methods

### 4.1. Data Acquisition and Processing

Fragments Per Kilobase of transcript per Million (FPKM) of RNA sequence data of the HCC were obtained from TCGA database [Project ID = “TCGA-LIHC” (https://portal.gdc.cancer.gov/ accessed on 1 October 2023)], including 371 HCC tissues and 50 adjacent normal tissues. HCC was defined as 141 HCC tissues and 21 adjacent normal tissues with overall survival greater than disease-free survival. All FPKM data were transformed into transcripts per million and normalized. The mRNAsi and EREG-mRNAsi scores were obtained using OCLR algorithm, which was published by Malta et al. [19]. The immune scores, stromal scores, and estimate scores were calculated by applying the ESTIMATE algorithm, where R package = estimate.

### 4.2. Mutation Analysis

Simple Nucleotide Variation data from TCGA database (project = TCGA-LIHC) were analyzed using R package “maftools” (v2.8.0). We performed comprehensive mutation profiling, including mutation frequency calculation, classification into categories (e.g., missense, nonsense), and spectrum analysis. TMB was calculated as non-synonymous mutations per megabase. An oncoplot visualized the mutation landscape across samples for the top 30 mutated genes. Co-occurrence and mutual exclusivity of gene mutations were assessed using pairwise Fisher’s exact tests. To correlate with stemness, samples were divided into high and low TMB groups based on median TMB, and mRNAsi values were compared between groups using Wilcoxon rank-sum test. All analyses used R (v4.0.3), with *p* < 0.05 considered significant.

### 4.3. DEGs of HCC

The DEGs between HCC and adjacent normal liver tissues in HCC were analyzed using the “FindMarkers” in the R package. In WGCNA, the cut-off thresholds employed for identifying DEGs were |log2 fold change| > 1 and *p* value < 0.05. In CSC genes selection, the cut-off thresholds were |log2 fold change| > 2 and *p*-value < 0.05.

### 4.4. WGCNA

The “WGCNA” in the R package was used to construct the co-expression network of the DEGs in HCC. We chose 6 as the optimal soft threshold (power) to enhance matrix similarity and construct a co-expression network. The module–trait correlations with mRNAsi and EREG-mRNAsi were constructed by R software. We chose the two modules with the highest mRNAsi correlation, ran correlation analyses on each, and screened for key genes associated with the module and the mRNAsi index. Key genes were intersected with DEGs (|log2 fold change| > 2 and *p* vaule < 0.05) to narrow down the candidates of CSC genes.

### 4.5. The Kaplan–Meier Survival Analysis

In the Kaplan–Meier survival analysis section, we evaluated the impact of mRNAsi values on the overall survival of HCC patients. The Kaplan–Meier method was employed to generate survival curves, comparing patients with high and low mRNAsi scores. The log-rank test was used to assess statistical significance between the groups. The Kaplan–Meier survival analysis was evaluated using the “survival” and “surviminer” in R package.

### 4.6. Single-Sample Gene Set Enrichment Analysis

In the ssGSEA section, the “ssGSEA” in the R package was used to calculate the representative immune cell score for each sample. Gene expression data from TCGA were used as input, and immune cell reference datasets were obtained from the TISIDB database Version 4.0 (http://cis.hku.hk/TISIDB/ accessed on 2 November 2023). Each sample was scored based on the enrichment of immune cell-specific gene signatures, allowing us to assess the relative abundance of immune cell types in each tumor. These scores were then used to categorize the samples into distinct immune cell infiltration profiles. This approach provided a comprehensive overview of the immune landscape within the tumor microenvironment of HCC.

### 4.7. Enrichment Analysis of CSCs Genes and Protein–Protein Interaction Network

Gene ontology and KEGG pathway enrichment were performed to explore the biological functions and pathways associated with the identified CSC genes using the R packages “clusterProfiler”, focusing on biological processes, cellular components, and molecular functions. The PPI network was constructed using the Search Tool for Retrieval of Interacting Genes (STRING, https://string-db.org/ accessed on 2 November 2023, version11.0b) online database [47] to identify potential interactions among the CSC-related genes. The PPI network was visualized to highlight the key nodes and edges, allowing us to infer the functional connectivity and molecular mechanisms underpinning CSCs in HCC.

### 4.8. RT-PCR

This study included 20 individuals with HCC who underwent tumor excision (IRB number, 202201394B0). Clinical information of these 20 individuals is summarized in Supplemental Table S1. Tissue specimens from both HCC and adjacent normal tissues were obtained and stored using RNAprotect Tissue Reagent (Qiagen, Hilden, Germany). Total RNA was extracted from each tissue sample using miRNeasy Mini Kit (Qiagen) and reverse transcribed to cDNA using High-Capacity cDNA Reverse Transcription Kit (Applied Biosystems, Foster City, CA, USA). The gene expression level was assessed using Power SYBR Green PCR Master Mix (Applied Biosystems) and 7500 Real-Time PCR System (Applied Biosystems). The sequences of the primers used are listed in Supplemental Table S1.

### 4.9. Statistical Analysis

All results were presented as the median value with interquartile range in box plot. Nonparametric statistics were performed using the Wilcoxon or Kruskal–Wallis test. In RT-PCR, the results were presented as the mean ± standard error. Pairwise comparisons were performed using the Mann–Whitney test. The differences were considered significant at *p* < 0.05.

## 5. Conclusions

In summary, we established 10 key genes of the cell cycle pathway that are associated with tumor cell stemness and immune cell infiltration. This provides a new possibility for investigating the mechanism of tumor cell stemness and immune cell infiltration in HCC. However, this study was based on bioinformatics analysis results, and further experiments are needed to confirm how these genes regulate CSCs, which influence HCC prognosis.

## Figures and Tables

**Table 1:** The expression of 10 key genes in different pathological stages of HCC.

Gene	Stage I+II	Stage III	*p*-Value
	n = 16 (Median [Q1, Q3])	n = 4 (Median [Q1, Q3])	
*PRC1*	6.71 [3.31, 8.66]	18.61 [12.87, 24.48]	0.005
*DTL*	7.75 [2.37, 13.44]	31.78 [25.79, 36.07]	0.021
*TOP2A*	10.76 [2.80, 13.55]	27.94 [23.77, 30.31]	0.016
*NEK2*	4.80 [1.39, 10.95]	18.88 [12.66, 27.07]	0.028
*CDC6*	9.11 [4.03, 12.13]	30.80 [15.03, 57.51]	0.009
*HJURP*	6.99 [2.37, 10.26]	17.47 [10.17, 29.12]	0.036
*MCM2*	4.46 [2.46, 6.50]	9.80 [8.37, 11.20]	0.036
*KIFC1*	6.55 [2.94, 10.92]	18.34 [15.06, 23.55]	0.012
*MELK*	4.34 [2.15, 9.91]	17.70 [12.77, 25.05]	0.021
*FOXM1*	8.06 [2.58, 9.54]	15.32 [13.34, 17.82]	0.016

CDC6: cell division cycle 6; DTL: denticleless E3 ubiquitin protein ligase homolog; FOXM1: forkhead box M1; HJURP: Holliday junction recognition protein; KIFC1: kinesin family member C1; MCM2: minichromosome maintenance complex component 2; MELK: maternal embryonic leucine zipper kinase; NEK2: NIMA-related kinase 2; PRC1: protein regulator of cytokinesis 1; TOP2A: DNA topoisomerase II alpha.

## Data Availability

The original contributions presented in the study are included in the article/Supplementary Material, further inquiries can be directed to the corresponding authors.

## Supplementary Materials

The supporting information can be downloaded at https://www.mdpi.com/article/10.3390/ijms252211969/s1.
