# Supplementary material for: Identification of Crucial Cancer Stem Cell Genes Linked to Immune Cell Infiltration and Survival in Hepatocellular Carcinoma
Source: Int J Mol Sci. 2024 Nov 7;25(22):11969. doi: 10.3390/ijms252211969 (PMC11593742; doi:10.3390/ijms252211969)
Supplement: Supplementary file 1 [file ijms-25-11969-s001.zip › ijms-3222622-supplementary.pdf]

Targeted gene name		Sequence
PRC1	forward	5’- GGCCACCATTATGTCTGGGTCA -3’
	reverse	5’- GCTTGTCTCTGCTCCTGGCTA -3’
DTL	forward	5’- ACCCTGGCAACCTCCTACT -3’
	reverse	5’- CCTGGTTTCTCCTCTAAGCCTCTAT -3’
TOP2A	forward	5’-TGAAGATGAAGATTCATTGAAGACGCTTCG -3’
	reverse	5’-ACAATGGGAGTGATAAATTCCTCCAGAAA -3’
NEK2	forward	5’-TCAGGCGAATTCCATACCGTTACTC -3’
	reverse	5’-CTTCTCTCAAGATTTCTTCTTTGCTCGTC -3’
CDC6	forward	5’- TTCAATTCTGTGCCCGCAAAGT -3’
	reverse	5’- GGAATCAGAGGCTCAGAAGGTGATTTAC-3’
HJURP	forward	5’- CTTGATTTACTTCGACTCCAGTGCAACATA-3’
	reverse	5’- AAATCGGATTTCAATCTCCCTCTGACG-3’
MCM2	forward	5’- GCCCGCTACCTTTCATTCCG-3’
	reverse	5’- TGGATGTTGATCTGACGAGCCTTAT -3’
KIFC1	forward	5’- CAAGGAGTCCCACGTGCCTTAC -3’
	reverse	5’- TTCACCTTGGAGGCAAAGCG-3’
MELK	forward	5’- GGGTTATACACTGAAGTGTCAAACACAGTC-3’
	reverse	5’- ATACCTTGCAGCTAGATAGGATGTCTTCCA -3’
FOXM1	forward	5’-CCAACTCAGCCTCCAGGACT -3’
	reverse	5’- CTCCCGTTTCTGCTCGCAA-3’
h-GAPDH	forward	5’GAAGGTGAAGGTCGGAGTC-3’
	reverse	5’GAAGATGGTGATGGGATTTC-3’

Table S1. The primers sequences used in real-time quantitative polymerase chain reaction.

Supplement 1

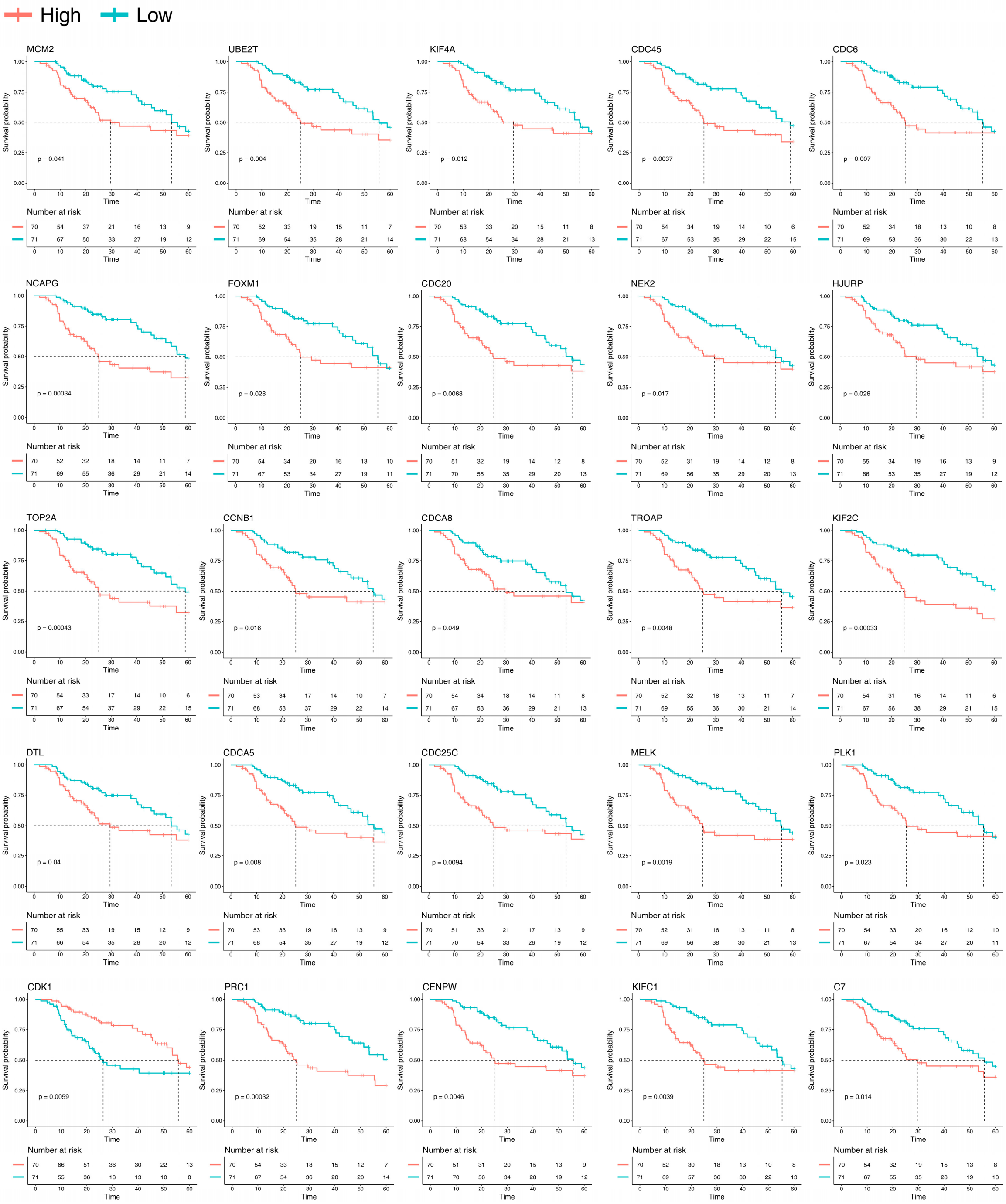

Figure S1. The Kaplan–Meier survival analysis. The figure presents the Kaplan–Meier survival analysis for 25 key genes associated with cancer stem cell characteristics in HCC. Each gene's impact on overall survival was assessed, revealing distinct survival curves for high and low expression groups.
